# Identifying and addressing obstacles to optimal alopecia areata and atopic dermatitis care in the UK

**DOI:** 10.1093/skinhd/vzag117

**Published:** 2026-07-28

**Authors:** Stephanie Gallard, Sue Schilling, Suzi Holland, Megan Mowbray, Sophia Conner, Manjit Kaur, Yousaf Aftab, Benoit Raymond, Gulraj Matharu, Karim Amer, Kave Shams, Leila Asfour

**Affiliations:** Dermatology GPSI for the Liverpool ICATS, Liverpool University Hospitals FT, Liverpool, UK; Alopecia UK, Shipley, UK; Eczema Outreach Support, Linlithgow, UK; Department of Dermatology, Queen Margaret Hospital, Dunfermline, UK; Department of Dermatology, West Glasgow Ambulatory Care Hospital, NHS Greater Glasgow and Clyde, Glasgow, UK; Department of Dermatology, Solihull Hospital, University Hospitals Birmingham NHS Foundation Trust, Solihull, UK; Pfizer Ltd, Walton Oaks, UK; Pfizer Ltd, Walton Oaks, UK; Pfizer Ltd, Walton Oaks, UK; Pfizer Ltd, Walton Oaks, UK; Dermatology Department, The Leeds Teaching Hospitals NHS Trust, Leeds, UK; Dermatology Department, Chelsea and Westminster NHS Foundation Trust, London, UK

## Abstract

Alopecia areata (AA) and atopic dermatitis (AD) are both chronic inflammatory conditions, associated with physical and significant psychosocial burden, with patients facing considerable barriers to care in the UK. In this perspective, we explore gaps in care in the UK healthcare system for patients with AA and AD, gaining insights from healthcare professionals, patients and patient advocates to identify opportunities for improvement within primary care and secondary care.

Patients with dermatological conditions face considerable barriers to accessing healthcare in the UK, as highlighted in the National Health Service (NHS) England 2021 Getting it Right First Time (GIRFT) national speciality report on dermatology.^1^ The GIRFT report highlighted inequities in access to and the variation in the availability of services, such as limited psychosocial support across dermatology care. Alopecia areata (AA) and atopic dermatitis (AD) are chronic, inflammatory conditions,^2–4^ associated with physical and significant psychosocial burden,^5–8^ with the burden exacerbated by the barriers to care.

In this perspective, we explore gaps in UK care for patients with AA and AD, drawing on insights from healthcare professionals, patients and patient advocates, to identify opportunities for improvement within primary care (PC) and secondary care (SC). Across a landscape assessment, patient and healthcare practitioner (HCP) interviews, a Delphi survey and an advisory board (detailed in the Supporting Information), patients with AA and AD were found to face system-wide gaps in care. However, clear steps can be taken to reduce these gaps and encourage systemic change.

## Access to secondary care was identified as a clear barrier for patients with alopecia areata and patients with atopic dermatitis

The long wait time between PC and SC was identified as a key barrier to timely patient care, supported by interviewed experts (reporting wait times from 3 months to 3 years), survey respondents (Tables 1, 2; Q43) and the 2023 NHS Dermatology report.^9^ Furthermore, geographical barriers to care were identified, with two of three patients with AA having to travel significant distances to get appropriate care, supported by a high proportion of survey participants (Tables 1, 2; Q40).

**Table 1:** Results from survey respondents on different aspects of care for alopecia areata (AA)

Ref.	Question/statement	Round of consensus	Response, % (*n*)			
			Disagree	Neutral	Agree	N/A
Q7	The level of education and training provided to GPs and other primary healthcare professionals is sufficient for diagnosing and optimally supporting patients with AA	3 (no consensus)	64 (28)	23 (10)	14 (6)	0 (0)
Q8	The current management in primary care is as effective as it could be for patients with AA	2	73 (42)	9 (5)	18 (10)	0 (0)
Q14	The introduction of standardized quick guides/checklists for GPs could improve primary care management of patients with AA	1	3 (2)	5 (5)	92 (89)	0 (0)
Q15	More comprehensive clinical guidelines (e.g. from the BAD and/or NICE, including for specific patient populations) could improve primary care management of patients with AA	1	3 (3)	3 (3)	95 (91)	0 (0)
Q17	GPs prioritize the management of patients with AA	3 (no consensus)	41 (18)	41 (18)	18 (8)	0 (0)
Q40	There are no differences in access to treatment based on ­geographical location for patients with AA	3 (no consensus)	55 (24)	23 (10)	9 (7)	14 (6)
Q43	The waiting times from GP referral to general dermatology appointments can negatively impact access to appropriate treatment management options for patients with AA	1	5 (5)	3 (3)	92 (88)	0 (0)
Q45	Available funding and resources for dermatology services within the NHS negatively impact the management options available for patients with AA	1	0 (0)	6 (5)	94 (91)	0 (0)
Q50	Timely access to psychosocial support services is readily available for patients with AA	2	73 (41)	9 (5)	5 (3)	5 (3)

The number of survey respondents dropped off with each subsequent round of review: round 1, *n* = 96; round 2, *n* = 57; round 3, *n* = 44. No survey questions were mandatory; thus, the number of responses varies for some questions. BAD, British Association of Dermatologists; GP, general practitioner; N/A, not applicable; NHS, National Health Service; NICE, National Institute of Health and Care Excellence.

**Table 2:** Results from survey respondents on different aspects of care for atopic dermatitis (AD)

Ref.	Question or statement	Round of consensus	Response, % (*n*)			
			Disagree	Neutral	Agree	N/A
Q7	The level of education and training provided to GPs and other primary healthcare professionals is sufficient for diagnosing and optimally supporting patients with AD	3 (no consensus)	27 (12)	18 (8)	55 (24)	0 (0)
Q8	The current management in primary care is as effective as it could be for patients with AD	3 (no consensus)	32 (14)	18 (8)	50 (22)	0 (0)
Q14	The introduction of standardized quick guides/checklists for GPs could improve primary care management of patients with AD	1	3 (2)	8 (7)	90 (86)	0 (0)
Q15	More comprehensive clinical guidelines (e.g. BAD and/or NICE, including for specific patient populations) could improve primary care management of patients with AD	1	3 (2)	3 (2)	95 (91)	0 (0)
Q17	GPs prioritize the management of patients with AD	3 (no consensus)	14 (6)	45 (20)	41 (18)	0 (0)
Q40	There are no differences in access to treatment based on ­geographical location for patients with AD	3 (no consensus)	59 (26)	23 (10)	5 (2)	14 (6)
Q43	The waiting times from GP referral to general dermatology appointments can negatively impact access to appropriate treatment management options for patients with AD	1	5 (5)	3 (2)	93 (89)	0 (0)
Q45	Available funding and resources for dermatology services within the NHS negatively impact the management options available for patients with AD	1	6 (5)	8 (8)	86 (83)	0 (0)
Q50	Timely access to psychosocial support services is readily available for patients with AD	3 (no consensus)	68 (30)	18 (8)	9 (4)	5 (2)

The number of survey respondents dropped off with each subsequent round of review: round 1, *n* = 96; round 2, *n* = 57; round 3, *n* = 44. No survey questions were mandatory; thus, the number of responses varies for some questions. BAD, British Association of Dermatologists; GP, general practitioner; N/A, not applicable; NHS, National Health Service; NICE, National Institute of Health and Care Excellence.

More dermatology specialists (e.g. clinicians and advanced nurse practitioners/pharmacist practitioners) are required to ensure better provision and equity of care across the UK. In the interim, it is important to establish clear and consistent standards for AA and AD management. This could be assisted using checklists, guides, the provision of accredited resources [e.g. British Association of Dermatologists (BAD), Primary Care Dermatology Society (PCDS)] (Tables 1, 2; Q14) and comprehensive clinical guidelines (Tables 1, 2; Q15) to ensure that the information given to patients with AA and AD is up-to-date best practice.

PC providers should be encouraged to refer patients with more severe, atypical and rapidly progressing AA, with marked psychological impact, to SC, enabling advice and guidance to be offered while waiting for an appointment in SC. Patients with AD should be referred as per their management plan. PC and SC providers should collaborate, crucially listening to the patient voice throughout their care.

## Patients need more support in primary care while waiting for a secondary care appointment

More support is required for patients with AA and/or AD in PC; however, general practitioners (GPs) are limited by time and money. Survey respondents indicated that current management of AA is ineffective (Table 1; Q8), supported by all (*n* = 3/3) patients with AA who had no management plan, reporting significant wait times and capacity issues in line with five of seven HCPs. For AD, there was no consensus on whether the current management in PC is as effective as it could be (Table 2; Q8). Furthermore, there was no agreement on whether GPs prioritize the care of patients with AA or AD (Tables 1, 2; Q17).

Integrating care through the use of pharmacists, often the first point of contact for patients with visible skin conditions, has been illustrated in the NHS long-term plan.^10^ This included the expansion of clinical pharmacists in general practice, and the pharmacy-­first service, enabling pharmacists to support patients, treat minor ailments and prescribe for certain conditions.

## Improving the knowledge around alopecia areata and atopic dermatitis in primary care is essential

The education of PC providers could be improved, as mentioned in the GIRFT report, in which dermatology training was said to be limited to 0–2 weeks over 5 years of training.^1^ English HCPs (*n* = 5/5) noted a lack of GP knowledge around AA, supported by survey participants (Table 1; Q7); however, this was not reflected by Scottish HCPs (*n* = 2/2). For AD, HCPs (*n* = 5/7) and survey respondents (Table 2; Q7) conveyed that GPs had a reasonable awareness of the condition, agreeing with patients (*n* = 2/2).

Signposting to existing resources, such as those available through the BAD, PCDS or Red Whale (see Supporting Information), rather than generating new resources, was recommended. Furthermore, the provision of easy-to-use checklists and short guides were suggested (Tables 1, 2; Q14) to help PC providers ensure the information they give to patients with AA and/or AD is the current best practice.

Improving the level of education of patients with AA and/or AD could improve their outcomes and empower patients to take charge of their care. Providing short information guides on their condition would allow patients to make informed care decisions and self-advocate to their GP while waiting for an SC appointment.

## Psychosocial support requires significant improvement

There was clear evidence that the psychosocial effect of AA and/or AD was undertreated within all aspects of this study. No patients (*n* = 0/5) received psychosocial support as part of their AA or AD management; only one of seven HCPs reported access to in-house, timely, psychosocial support, further evidenced by survey participants (Tables 1, 2; Q50).

Acknowledging the burden on patients with AA and/or AD and how they are feeling is an easy first step that can be provided across all levels of care. Providing patients with self-help materials and support from dedicated charities were identified as valuable tools that could be used across PC and SC, although certain patients may require targeted psychological care. Patient ­wellbeing should be evaluated using validated tools, such as patient-­reported outcome measures and quality of life surveys. Furthermore, basic psychodermatology training could be provided to SC providers to ensure that they can better identify, manage and support patients who need this specialist help and broader training to ensure that psychosocial tools and skills are used during consultations.

## Bureaucratic barriers affect the availability of the treatment

The availability of funding and resources were seen to affect the care for patients with AA and AD (Tables 1, 2; Q45), with administrative challenges required to set up delivering new medications within the health service highlighted. The approval of new cost-­effective treatments by the National Institute for Health and Care Excellence or the Scottish Medicines Consortium should result in a statutory requirement for treatment availability to eligible patients; however, two of seven HCPs reported lacking access to such approved treatments, with a further two noting a lack of support in making such treatments available. Supporting and advising commissioning teams within the Trusts on the implementation of new treatments to reduce the variations in access would help to minimize the burden on patients and clinicians treating dermatological conditions like AA and/or AD.

## Conclusion

System-wide changes are required to improve outcomes and management of care for UK patients with AA and/or AD. Simple, focused changes could be implemented, including improving education on AA and/or AD management, greater collaboration between PC and SC, equitable access to psychology services, optimizing pathways and digital innovation to reduce NHS operational burdens. Additional research may be required to understand the best approach, especially with the upcoming structural and operational changes to the NHS England structure.

## Supplementary Material

vzag117_Supplementary_Data

## Data Availability

Full details obtained from the survey respondents, including respondents by job role, location of respondents and results from each round of the survey, are available in the Supporting Information. The data that support the findings of this study are available from Pfizer UK Ltd; restrictions may apply to the availability of these data. Data are available from the authors with the permission of Pfizer UK Ltd.
