# Supplementary material for: Identifying and addressing obstacles to optimal alopecia areata and atopic dermatitis care in the UK
Source: Skin Health Dis. 2026 Jul 28;6(5):838–42. doi: 10.1093/skinhd/vzag117 (PMC13623443; doi:10.1093/skinhd/vzag117)
Supplement: Supplementary file 1 [file vzag117_supplementary_data.docx]

# Identifying and addressing obstacles to optimal Alopecia Areata and Atopic Dermatitis care in the UK

Stephanie Gallard BSc (Hons) Pharmacy, MBCHB, Dip Prac Derm, MSc,^1^ Sue Schilling PGCert,^2^ Suzi Holland MA (Hons),^3^ Megan Mowbray MBChB, BSc (Hons), FRCP, MD (Derm),^4^ Sophia Conner MPharm MSc (IP),^5^ Manjit R Kaur BMedSci (Hons), BMBS, FRCP, PGCMedEd, CCT,^6^ Yousaf Aftab MSC MPharms (IPresc) CMD,^7^ Benoit Raymond PhD,^7^ Gulraj S. Matharu MBChB, DPhil,^7^ Karim Amer MD, MBA,^7^ Kave Shams MD,^8^ and Leila Asfour MBChB BSc MRCP (Derm)^9^*

*Corresponding author

1. Liverpool University Hospitals FT, c/o Greenbank Road Surgery, Liverpool, Merseyside, L18 1HG, UK
2. Alopecia UK, PO Box 341, Shipley, BD18 9EH, UK
3. Eczema Outreach Support, Linlithgow, EH49 7EZ, UK
4. Department of Dermatology, Queen Margaret Hospital, Whitefield Road, Dunfermline, KY12 0SU
5. West Glasgow Ambulatory Care Hospital, NHS Greater Glasgow and Clyde, Glasgow, G3 8SJ, UK
6. Department of Dermatology, Solihull Hospital, University Hospitals Birmingham NHS Foundation Trust, Solihull, B91 2JL, UK
7. Pfizer Ltd, Walton Oaks, Surrey, KT20 7NS, UK
8. Dermatology Department, The Leeds Teaching Hospitals NHS Trust, Leeds, LS7 4SA, UK
9. Dermatology Department, Chelsea and Westminster NHS Foundation Trust, London, SW10 9NH, UK

**Supporting Information**

Contents

[List of abbreviations	3](#_Toc230785058)

[Links to suggested resources	4](#_Toc230785059)

[S1 1: Methodology	5](#_Toc230785060)

[SI 1.1 Landscape assessment	5](#_Toc230785061)

[SI 1.2 Delphi consensus survey	5](#_Toc230785062)

[SI 1.3 Advisory board meeting and discussion	5](#_Toc230785063)

[SI 2: Landscape assessment	6](#_Toc230785064)

[SI 2.1: Interview questions for the HCPs	6](#_Toc230785065)

[SI 2.2: Interview questions for patients with AA/AD	6](#_Toc230785066)

[SI 3: Delphi survey	7](#_Toc230785067)

[SI 3.1: Respondents in each round by job role	7](#_Toc230785068)

[SI 3.2: Location of Delphi respondents	7](#_Toc230785069)

[SI 3.3: Delphi survey questions and results	8](#_Toc230785070)

[SI: 3.3.1: Complete results of the Delphi survey for questions relating to AA, including responses from each survey round	8](#_Toc230785071)

[SI: 3.3.2: Complete results of the Delphi survey for questions relating to AD, including responses from each survey round	19](#_Toc230785072)

# List of abbreviations

AA – alopecia areata

AD – atopic dermatitis

BAD – British Association of Dermatologists

BHNS – British Hair and Nail Society

EOS – Eczema outreach support

GP – general practitioner

HCP – healthcare practitioner

ICB – Integrated care board

JAKi – Janus kinase inhibitor

NICE – National Institute of Health and Care Excellence

NICE TA – NICE Technology Appraisal

PAG – Patient advisory group

PCDS – Primary Care Dermatology Society

RWE – Real-world evidence

SMC – Scottish Medicines Consortium

# Links to suggested resources

Red whale <https://www.redwhale.co.uk/>

Primary Care Dermatological Society. Alopecia areata. <https://www.pcds.org.uk/clinical-guidance/alopecia-areata>

Primary Care Dermatological Society. Atopic eczema. <https://www.pcds.org.uk/clinical-guidance/atopic-eczema>

Primary Care Dermatology Society. National primary care treatment and referral guidelines for common skin conditions. <https://www.pcds.org.uk/desktop-treatment-guide>

The British Association of Dermatology topical corticosteroid guide <https://www.bad.org.uk/pils/topical-corticosteroids>

The British Association of Dermatology alopecia areata patient information leaflet <https://www.bad.org.uk/pils/alopecia-areata>

The British Association of Dermatology atopic eczema patient information leaflet <https://www.bad.org.uk/pils/atopic-eczema>

Eczema written action plan: <http://www.bristol.ac.uk/primaryhealthcare/researchthemes/apache/ewap/>

British Association of Dermatologists. Patient management pathway for people with alopecia areata. <https://cdn.bad.org.uk/uploads/2021/12/19201608/Patient-Management-Pathway.pdf>

NHS Scotland. Dermatology pathways | right decisions. <https://www.rightdecisions.scot.nhs.uk/dermatology-pathways/>

Royal College of General Practitioners. Course: clinical toolkits | RCGP learning. <https://elearning.rcgp.org.uk/course/view.php?id=537&expandsection=0#section-0>

National Institute for Health and Care Excellence. Atopic eczema in under 12s: diagnosis and management. <https://www.nice.org.uk/guidance/cg57/resources>

BNF information on topical corticosteroids <https://bnf.nice.org.uk/treatment-summaries/topical-corticosteroids/>

BAD clinical guidance <https://www.bad.org.uk/guidelines-and-standards/clinical-guidelines>

# S1 1: Methodology

A mixed-methods study was conducted, divided into three stages: a landscape assessment, Delphi survey, and an advisory board meeting. The information collected at each stage was used iteratively to inform the next stage.

## SI 1.1 Landscape assessment

The landscape assessment looked at the management of AA and AD, associated challenges, and potential educational requirements. This was completed through targeted narrative literature review research, conducted on PubMed, and through interviews with 7 secondary and tertiary healthcare professionals (4 consultant dermatologists and 3 specialist dermatology pharmacists), 3 patients with AA and 2 patients with AD. Participants were asked 8 open-ended questions in 30 minute 1:1 virtual interviews (**SI 2.1 and SI 2.2**), which were then analysed thematically to generate survey questions for a Delphi consensus.

## SI 1.2 Delphi consensus survey

The online Delphi survey questionnaire was circulated to opted-in UK healthcare professional (HCP) subscribers working in primary and secondary care through Cogora media channels. This included opt-in emails and newsletters to Pulse, Hospital Healthcare Europe, Healthcare Leader, and Hospital Pharmacy Europe subscribers.

Prior to the survey being distributed, threshold consensus of any question was set at ≥70% with a total number of 3 survey rounds (R). Questions that reached consensus were not included in the following survey round. Respondents received a copy of the responses from each round with the next round of the survey. Survey responses were analysed for consensus trends. A breakdown of the respondents by round, job role (**SI 3.1**) and their location (**SI 3.2**) are available in the supplementary information.

## SI 1.3 Advisory board meeting and discussion

Virtual 1:1 interviews were conducted with advisory board participants (7 HCPs; 4 consultant dermatologists, 2 specialist dermatology pharmacists and 1 GP with extended role in dermatology, and 2 patient advocacy group [PAG] leaders) to give feedback on the Delphi survey results. Four interviewees had not participated in the initial landscape assessment, so brought fresh perspectives to the discussion.

A half-day virtual advisory board meeting was held, evaluating the results from the landscape assessment, Delphi survey, and 1:1 interviews to provide further insights and discuss possible solutions to identified barriers to care. Break-out sessions were held, focusing on improving healthcare in the UK for AA and AD patients.

# SI 2: Landscape assessment

## SI 2.1: Interview questions for the HCPs

1. In your experience, what are the barriers to timely and accurate diagnosis of AA and/or AD? Who makes this diagnosis?
2. Can you describe how you think primary and secondary care interact in the management of AA and/or AD to ensure continuity of care?
3. In your experience, who are the key decision makers in determining the availability of treatment options for AA and/or AD?
4. Can you describe the treatment pathways for AA and/or AD as they currently stand?
5. What local/regional/national policies are you aware of that may have impacted your ability to treat patients with AA and/or AD? Can you please tell us about that?
6. In your opinion, what would optimal treatment pathways for patients with AA and/or AD look like?
7. What do you feel stands in the way of achieving this optimal AA/AD treatment pathway?
8. AA and AD can pose a significant burden for patients and their caregivers and families – in your opinion, what could be done to lessen this burden and improve access to timely management?

## SI 2.2: Interview questions for patients with AA/AD

1. Who made your AA/AD diagnosis, and did you feel like it was straightforward to receive?
2. When you go to the GPs, do you feel like they know what is going on with your AA/AD?
3. What do you think determines which treatment options are available to you?
4. Can you walk us through your AA/AD treatment journey?
5. What changes (if any) can you think of that may have altered your management plan for AD/AA?
6. If there were no barriers (like cost, staff, NHS rules), what would an optimal AA/AD treatment plan look like for you?
7. What do you think is stopping us from having that system?
8. Having AA/AD can be stressful for patients and their families – what could be done to better support you and your family?

# SI 3: Delphi survey

## SI 3.1: Respondents in each round by job role

| **Role Description** | **Respondents, % (n)** | | |
| --- | --- | --- | --- |
|  | **Round 1, N=97** | **Round 2,**  **N=57** | **Round 3,**  **N=44** |
| General Practitioner | 53.13 (52) | 61.40 (35) | 63.64 (28) |
| General Practitioner with a Special Interest | 9.38 (9) | 10.53 (6) | 9.09 (4) |
| Secondary Care Physician | 6.25 (6) | 7.02 (4) | 9.09 (4) |
| Hospital Pharmacist | 14.58 (14) | 14.04 (8) | 11.36 (5) |
| Community Pharmacist | 9.38 (9) | 3.51 (2) | 4.55 (2) |
| Commissioner | 2.08 (2) | 0.00 (0) | 0.00 (0) |
| Commissioning Supporting Role | 3.13 (3) | 1.75 (1) | 2.27 (1) |
| Primary Care Leader | 2.08 (2) | 1.75 (1) | 0.00 (0) |

## SI 3.2: Location of Delphi respondents

| **Location** | **%Respondents** | | |
| --- | --- | --- | --- |
|  | **Round 1,**  **N=97** | **Round 2,**  **N=57** | **Round 3,**  **N=44** |
| London | 14.55 | 17.54 | 9.09 |
| North West | 12.73 | 22.81 | 18.18 |
| West Midlands | 12.73 | 17.54 | 13.64 |
| South West | 10.91 | 7.02 | 18.18 |
| East of England | 10.91 | 10.53 | 9.09 |
| Yorkshire | 7.27 | 5.26 | 9.09 |
| East Midlands | 7.27 | 5.26 | 4.55 |
| South East | 5.45 | 3.51 | 4.55 |
| North East | 3.64 | 0.00 | 0.00 |
| West of Scotland | 3.64 | 1.75 | 2.27 |
| Central Scotland | 1.82 | 3.51 | 2.27 |
| Glasgow | 1.82 | 1.75 | 0.00 |
| Highlands | 1.82 | 0.00 | 0.00 |
| Antrim | 1.82 | 1.75 | 2.27 |
| Down | 1.82 | 1.75 | 2.27 |
| Fermanagh | 1.82 | 0.00 | 0.00 |

## SI 3.3: Delphi survey questions and results

### SI: 3.3.1: Complete results of the Delphi survey for questions relating to AA, including responses from each survey round

**Table 3.3.1.1:** A summary of the key consensus results from the Delphi survey for AA, including all rounds of responses.

| **Ref** | **Question/statement** | **Round** | **Response % (n)** | | | |
| --- | --- | --- | --- | --- | --- | --- |
|  |  |  | **Disagree** | **Neutral** | **Agree** | **N/A** |
| **Q6** | General awareness of GPs and other primary care professionals is high regarding the signs and symptoms of AA | 1  2  3 | 32.5 (31)  36.36 (21)  31.82 (14) | 12.50 (12)  36.36 (21)  22.73 (10) | 55.00 (53)  27.27 (16)  54.55 (24) | 0 (0)  0 (0)  0 (0) |
| **Q7** | The level of education and training provided to GPs and other primary healthcare professionals is sufficient for diagnosing and optimally supporting patients with AA | 1  2  3 | 45.00 (43)  59.09 (34)  63.64 (28) | 35.00 (34)  22.73 (13)  22.73 (10) | 20.00 (19)  18.18 (17)  13.64 (6) | 0 (0)  0 (0)  0 (0) |
| **Q8** | The current management in primary care is as effective as it could be for patients with AA | 1  2 | 63.41 (61)  72.7 (42) | 21.95 (21)  9.09 (5) | 14.63 (14)  18.18 (10) | 0 (0)  0 (0) |
| **Q9** | Quality disease-specific information is consistently provided by GPs to patients on possible management options and outcomes, and information on next steps for patients with AA | 1  2  3 | 57.5 (55)  50 (29)  68.18 (30) | 25 (24)  36.36 (21)  13.64 (6) | 17.50 (17)  18.18 (17)  18.18 (8) | 0 (0)  0 (0)  0 (0) |
| **Q10** | GPs appropriately refer patients to secondary care with AA | 1  2  3 | 12.82 (12)  31.82 (31)  27.27 (12) | 23.08 (22)  22.73 (22)  22.73 (10) | 61.54 (59)  45.45 (44)  50.00 (22) | 2.56 (2)  0 (0)  0 (0) |
| **Q11** | GP referral to secondary care is in general timely for patients with AA | 1  2  3 | 40.00 (38)  45.45 (26)  40.91 (18) | 27.50 (26)  31.82 (18)  13.64 (6) | 30.00 (29)  22.73 (21)  45.45 (20) | 2.50 (2)  0 (0)  0 (0) |
| **Q12** | The use of the Advice and Guidance (A&G) teledermatology portal by GPs improves the management and the process of referral to secondary care for patients with AA | 1  2  3 | 15.00 (14)  18.18 (10)  22.73 (10) | 15.00 (14)  31.82 (18)  18.18 (8) | 57.50 (55)  45.45 (26)  59.09 (26) | 12.50 (12)  9.09 (5)  0 (0) |
| **Q13** | The use of the Advice and Guidance (A&G) teledermatology portal by GPs may result in over-reliance of secondary care for conditions that could in many cases be successfully managed in primary care alone, such as AA | 1  2  3 | 41.03 (39)  31.82 (18)  54.55 (24) | 23.08 (22)  18.18 (10)  18.18 (8) | 28.21 (27)  45.45 (26)  27.27 (12) | 7.69 (7)  4.55 (3)  0 (0) |
| **Q14** | The introduction of standardised quick guides/checklists for GPs could improve primary care management of patients with AA | 1 | 2.56 (2) | 5.13 (5) | 92.31 (89) | 0 (0) |
| **Q15** | More comprehensive clinical guidelines (e.g., from the British Association of Dermatologists (BAD) and/or National Institute of Health and Care Excellence (NICE), including for specific patient populations) could improve primary care management of patients with AA | 1 | 2.63 (3) | 2.63 (3) | 94.74 (91) | 0 (0) |
| **Q17** | GPs prioritise the management of patients with AA | 1  2  3 | 35.90 (34)  45.45 (26)  40.91 (18) | 41.03 (39)  36.36 (21)  40.91 (18) | 23.08 (22)  18.18 (10)  18.18 (8) | 0 (0)  0 (0)  0 (0) |
| **Q19** | There is a high level of awareness and knowledge among general dermatologists (e.g., non-tertiary hair specialists) around all approved medications and their appropriate use in patients with AA | 1  2  3 | 16.22 (16)  13.64 (8)  22.73 (10) | 21.62 (21)  22.73 (13)  13.64 (6) | 40.54 (39)  50.00 (29)  50.00 (22) | 21.62 (21)  4.55 (3)  13.64 (6) |
| **Q20** | There is a high level of understanding of all available management strategies and their appropriate application by general dermatologists for patients with AA | 1  2  3 | 13.51 (13)  4.55 (3)  22.73 (10) | 16.22 (16)  27.27 (16)  13.64 (6) | 48.65 (47)  54.55 (31)  50.00 (22) | 21.62 (21)  4.55 (3)  18.18 (8) |
| **Q21** | There is a high level of education and training provided to general dermatologists on recognising and managing AA | 1  2  3 | 10.81 (10)  9.09 (5)  13.64 (6) | 13.51 (13)  50.00 (29)  22.73 (10) | 48.65 (47)  31.82 (18)  45.45 (20) | 27.03 (26)  4.55 (3)  13.64 (6) |
| **Q22** | Comprehensive and complete management is provided in secondary care for patients with AA | 1  2  3 | 8.57 (8)  22.73 (13)  22.73 (10) | 28.57 (27)  28.57 (16)  27.27 (10) | 40.00 (38)  45.45 (26)  31.82 (14) | 22.86 (22)  22.86 (13)  18.18 (8) |
| **Q23** | Comprehensive and complete access to care is available in secondary care for patients with | 1  2  3 | 21.62 (21)  27.27 (16)  50.00 (22) | 24.32 (32)  31.82 (18)  13.64 (6) | 32.43 (31)  27.27 (16)  18.18 (8) | 21.62 (21)  4.55 (3)  18.18 (8) |
| **Q24** | Currently available management options provided in secondary care are satisfactory for patients with AA | 1  2  3 | 29.73 (29)  31.82 (18)  40.91 (18) | 29.73 (29)  18.18 (10)  13.64 (6) | 24.32 (23)  36.36 (21)  22.73 (10) | 16.22 (16)  4.55 (3)  18.18 (8) |
| **Q25** | Although not life-threatening, clinicians prioritise management of conditions with life-limiting symptoms, such as AA | 1  2  3 | 31.58 (30)  27.27 (16)  40.91 (18) | 31.58 (30)  36.36 (21)  18.18 (8) | 23.68 (23)  22.73 (13)  22.73 (10) | 13.16 (13)  4.55 (3)  18.18 (8) |
| **Q26** | Quality disease-specific information is consistently provided by dermatologists to patients on possible management options and outcomes, and information on next steps for patients with AA | 1  2  3 | 18.92 (18)  4.55 (3)  18.18 (8) | 43.24 (25)  36.36 (21)  27.27 (12) | 16.22 (16)  45.45 (26)  36.36 (16) | 21.62 (21)  4.55 (3)  18.18 (8) |
| **Q27** | Patient-reported outcome measures are routinely used in secondary care to assess the appropriateness and success of treatments in patients with AA | 1  2  3 | 16.28 (16)  4.55 (3)  22.73 (10) | 23.26 (23)  45.45 (26)  18.18 (8) | 41.86 (40)  31.82 (18)  36.36 (16) | 18.60 (18)  9.09 (5)  22.73 (10) |
| **Q28** | Quality of life (QoL) measures/indexes are routinely used to help make treatment decisions and determine the impact on patient wellbeing for patients with AA | 1  2  3 | 11.11 (11)  13.64 (8)  36.36 (16) | 30.56 (29)  40.91 (23)  18.18 (8) | 11.11 (11)  27.27 (16)  27.27 (10) | 22.22 (21)  9.09 (5)  18.18 (8) |
| **Q30** | The guidance supplied by NICE is sufficiently comprehensive for managing patients with AA | 1  2  3 | 5.56 (5)  18.18 (10)  27.27 (12) | 61.11 (59)  36.36 (21)  31.82 (14) | 25.00 (24)  27.27 (16)  31.82 (14) | 8.33 (8)  9.09 (5)  9.09 (4) |
| **Q31** | The guidance supplied by NICE is sufficiently up to date for managing patients with AA | 1  2  3 | 8.11 (8)  22.73 (13)  27.27 (12) | 51.35 (49)  51.35 (29)  31.82 (14) | 32.43 (31)  36.36 (21)  31.82 (14) | 8.11 (8)  8.11 (5)  9.09 (4) |
| **Q35** | Additional support or guidance beyond what is currently available in the UK is not required for managing patients with AA | 1  2  3 | 51.35 (49)  59.09 (34)  63.64 (28) | 32.43 (31)  13.64 (8)  13.64 (6) | 10.81 (10)  18.18 (10)  18.18 (8) | 5.41 (5)  0 (0)  4.55 (2) |
| **Q37** | Treatment pathways are largely identical in different UK regions across the NHS for patients with AA | 1  2  3 | 26.47 (25)  22.73 (13)  27.27 (12) | 26.47 (25)  36.36 (21)  36.36 (16) | 35.29 (34)  18.18 (10)  18.18 (8) | 11.76 (11)  13.64 (8)  18.18 (8) |
| **Q38** | National guidelines (e.g., NICE guidelines or NICE technology appraisals) impact significantly on the availability and use of management options for AA | 1  2  3 | 8.33 (8)  9.09 (5)  4.55 (2) | 33.33 (32)  22.73 (13)  22.73 (10) | 58.33 (56)  59.09 (34)  59.09 (26) | 0 (0)  0 (0)  13.64 (6) |
| **Q39** | Following publication of NICE technology appraisals or Scottish Medicines Consortium (SMC) recommendations, there is timely implementation of new management options for patients with AA | 1  2  3 | 29.73 (29)  31.82 (18)  40.91 (18) | 45.95 (44)  13.64 (8)  27.27 (12) | 8.11 (8)  27.27 (16)  13.64 (6) | 16.22 (16)  18.18 (10)  18.18 (8) |
| **Q40** | There are no differences in access to treatment based on geographical location for patients with AA | 1  2  3 | 52.78 (51)  40.91 (23)  54.55 (24) | 33.33 (32)  22.73 (13)  22.73 (10) | 5.56 (5)  18.18 (10)  9.09 (7) | 8.33 (8)  9.09 (5)  13.64 (6) |
| **Q41** | The Quality and Outcomes Framework should set out target indicators that can be met in the management of patients with AA | 1  2  3 | 22.22 (21)  27.27 (16)  22.73 (10) | 30.56 (29)  27.27 (16)  27.27 (12) | 38.89 (37)  31.82 (18)  36.36 (16) | 8.33 (8)  4.55 (3)  13.64 (6) |
| **Q42** | The requirement to be seen in general dermatology clinics prior to a specialist dermatology clinic (if available, e.g., hair specialist clinic) can negatively impact the management of patients with AA | 1  2  3 | 19.44 (19)  27.27 (16)  13.64 (6) | 22.22 (21)  22.73 (13)  31.82 (14) | 58.33 (56)  40.91 (23)  40.91 (18) | 0 (0)  4.55 (3)  13.64 (6) |
| **Q43** | The waiting times from GP referral to general dermatology appointments can negatively impact access to appropriate treatment management options for patients with AA | 1 | 5.41 (5) | 2.70 (3) | 91.9 (88) | 0 (0) |
| **Q44** | The waiting times from secondary care general dermatology to tertiary care specialist appointments (e.g. tertiary hair specialists, or tertiary psychodermatology services) can negatively impact access to appropriate management options for patients with | 1 | 0 (0) | 5.41 (5) | 83.78 (80) | 10.81 (10) |
| **Q45** | Available funding and resources for dermatology services within the NHS negatively impact the management options available for patient with AA | 1 | 0 (0) | 5.56 (5) | 94.44 (91) | 0 (0) |
| **Q46** | Current UK public health policies and priorities limit the optimal management of AA | 1  2  3 | 5.56 (5)  9.09 (5)  4.55 (2) | 22.22 (21)  22.73 (13)  18.18 (8) | 63.89 (61)  50.00 (59)  59.09 (26) | 2.78 (3)  9.09 (5)  18.18 (8) |
| **Q47** | There is a timely and widespread access to specialist expertise in AA | 1  2  3 | 63.89 (61)  54.55 (31)  45.45 (20) | 13.89 (13)  27.27 (16)  18.18 (8) | 22.22 (21)  9.09 (5)  18.18 (8) | 0 (0)  0 (0)  13.64 (6) |
| **Q50** | Timely access to psychosocial support services is readily available for patients with AA | 1  2 | 67.57 (30)  72.70 (41) | 18.92 (11)  9.09 (5) | 8.11 (8)  4.55 (3) | 5.41 (5)  4.55 (3) |
| **Q52** | Established management pathway models, such as for psoriasis, should be adapted and implemented for an optimal patient journey for patients with AA | 1 | 10.53 (10) | 10.53 (10) | 73.69 (71) | 5.26 (5) |
| **Q53** | A national patient management pathway that clearly identifies all available management options and the process for selecting, initiating and monitoring is needed for patients with AA | 1 | 4.76 (5) | 19.05 (18) | 76.20 (73) | 0 (0) |
| **Q54** | A national patient management pathway that clearly identifies approaches and guidance for patients having an inadequate or suboptimal response to their treatment with instructions on appropriate alternative treatment options are needed for patients with AA | 1 | 4.55 (4) | 18.18 (17) | 77.27 (74) | 0 (0) |

**Table 3.3.1.2:** Results from Q16 of the Delphi survey, which asked whether participants were aware of incentives (e.g., financial, resource) for GPs with regards to the management of patients with AA

| **Ref** | **Round** | **Yes** | **No** |
| --- | --- | --- | --- |
| **Q16** | 1 | 0% | 100% |

**Table 3.3.1.3:** Results from Q18 of the Delphi survey, which asked, “Of all the topics listed in this section, which three do you feel are the most crucial to address to improve management of patients with AA?”

| **Ref** | **Topic** | **Round** | **%Responses (n)** |
| --- | --- | --- | --- |
| **Q18** | Increased GP awareness and training/education | 1  2  3 | 71.79 (69)  72.73 (41)  77.27 (34) |
|  | Provision of more patient information | 1  2  3 | 28.21 (27)  27.27 (16)  50.00 (22) |
|  | Increased referral to secondary care | 1  2  3 | 15.38 (15)  18.18 (10)  40.91 (18) |
|  | Increased use of teledermatology | 1  2  3 | 30.77 (30)  31.82 (18)  36.36 (16) |
|  | Additional tools for GPs | 1  2  3 | 30.77 (30)  31.82 (18)  27.27 (16) |
|  | Improved clinical guidelines and pathways | 1  2  3 | 82.05 (79)  72.73 (41)  72.73 (32) |
|  | More incentives for GPs (e.g. financial, resource) | 1  2  3 | 17.95 (17)  9.09 (5)  13.64 (6) |

**Table 3.3.1.4:** Summarised results from Q18 of the Delphi survey, which asked, “Of all the topics listed in this section, which three do you feel are the most crucial to address to improve management of patients with AA?”, identifying the top 3 crucial topics.

| **Ref** | **Round** | **Top 3 most crucial topics for AA** |
| --- | --- | --- |
| **Q18** | 1 | Improved clinical guidelines and pathways  Increased GP awareness and training/education  Increased use of teledermatology **and** Additional tools for GPs (tied) |
|  | 2 | Improved clinical guidelines and pathways **and** Increased GP awareness and training/education  Increased use of teledermatology **and** Additional tools for GPs |
|  | 3 | Increased GP awareness and training  Improved clinical guidelines and pathways  Provision of more patient information |

**Table 3.3.1.5:** Results from Q29 of the Delphi survey, which asked, “How familiar are you with the current NICE clinical guidelines and NICE technology appraisals for the treatment of AA?”

| **Ref** | **Round** | **%Responses (n)** | | | | | |
| --- | --- | --- | --- | --- | --- | --- | --- |
|  |  | **Not at all familiar** | **Slightly familiar** | **Somewhat familiar** | **Familiar** | **Very familiar** | **N/A to my practice** |
| **Q29** | 1  2  3 | 27.03 (26)  18.18 (10)  18.18 (8) | 13.51 (13)  22.73 (13)  22.73 (10) | 35.14 (34)  18.18 (10)  13.64 (6) | 13.51 (13)  18.18 (10)  36.36 (16) | 8.11 (8)  9.09 (5)  9.09 (4) | 2.70 (3)  4.55 (3)  4.55 (2) |

**Table 3.3.1.6:** Results from Q32 of the Delphi survey, which asked, “To what extent in your practice do you follow the British Association of Dermatologists (BAD) recommended treatment protocols and guidance for the management of patients with AA.”

| **Ref** | **Round** | **%Responses (n)** | | | | | |
| --- | --- | --- | --- | --- | --- | --- | --- |
|  |  | **Not at all** | **Rarely** | **Sometimes** | **Often** | **At all times** | **N/A to my practice** |
| **Q32** | 1  2  3 | 5.41 (5)  4.55 (3)  0.00 (0) | 13.51 (13)  13.64 (8)  4.55 (2) | 27.03 (26)  13.64 (8)  13.64 (6) | 37.84 (36)  50.00 (29)  63.64 (28) | 5.41 (5)  4.55 (3)  9.09 (4) | 10.81 (10)  4.55 (3)  9.09 (4) |

**Table 3.3.1.7:** Results from Q33 of the Delphi survey, which asked, “Do any of the following factors prevent you from being able to implement BAD management protocols for patients with AD, and if so, to what extent?”

|  | **Round** | **%Responses (n)** | | | | | |
| --- | --- | --- | --- | --- | --- | --- | --- |
|  |  | **Never** | **Rarely** | **Some-times** | **Often** | **Always** | **N/A to my practice** |
| Awareness | 1  2  3 | 11.36 (11)  10.00 (6)  9.09 (4) | 22.73 (22)  30.00 (17)  31.82 (14) | 34.09 (33)  40.00 (23)  36.36 (16) | 18.18 (17)  15.00 (9)  18.18 (8) | 9.09 (9)  5.00 (3)  0.00 (0) | 4.55 (4)  0.00 (0)  0.00 (0) |
| Confidence | 1  2  3 | 11.36 (11)  5.00 (3)  13.64 (8) | 31.82 (31)  35.00 (20)  31.82 (14) | 31.82 (31)  40.00 (23)  31.82 (14) | 15.91 (15)  15.00 (9)  18.18 (8) | 4.55 (4)  5.00 (3)  0.00 (0) | 4.55 (4)  0.00 (0)  4.55 (2) |
| Available management and treatment options in your region/local area | 1  2  3 | 0.00 (0)  0.00 (0)  4.55 (2) | 6.82 (7)  25.00 (14)  4.55 (2) | 38.64 (37)  15.00 (9)  36.36 (16) | 47.73 (46)  40.00 (23)  36.36 (16) | 2.27 (2)  20.00 (11)  13.64 (6) | 4.55 (4)  0.00 (0)  4.55 (2) |
| Practitioner/resource capacity | 1  2  3 | 0.00 (0)  0.00 (0)  4.55 (2) | 15.91 (15)  10.00 (10)  13.64 (8) | 36.36 (35)  20.00 (11)  31.82 (14) | 29.55 (28)  45.00 (26)  27.27 (12) | 13.64 (13)  25.00 (14)  18.18 (8) | 4.55 (4)  0.00 (0)  4.55 (2) |
| Patient choice | 1  2  3 | 2.27 (2)  10.00 (6)  4.55 (2) | 15.91 (15)  20.00 (11)  27.27 (12) | 56.82 (55)  20.00 (11)  50.00 (22) | 13.64 (13)  35.00 (20)  9.09 (4) | 4.55 (4)  10.00 (6)  4.55 (2) | 6.82 (7)  5.00 (3)  4.55 (2) |
| Implementation and adoption | 1  2  3 | 4.55 (4)  5.00 (3)  13.64 (6) | 25.00 (24)  20.00 (11)  4.55 (2) | 38.64 (37)  35.00 (20)  50.00 (22) | 20.45 (20)  20.00 (11)  22.73 (10) | 4.55 (4)  15.00 (9)  4.55 (2) | 6.82 (7)  5.00 (3)  4.55 (2) |
| Ease of use | 1  2  3 | 6.82 (7)  10.00 (6)  9.09 (4) | 18.18 (17)  15.00 (9)  22.73 (10) | 52.27 (50)  30.00 (17)  36.36 (16) | 11.36 (11)  30.00 (17)  27.27 (12) | 4.55 (4)  15.00 (9)  0.00 (0) | 6.82 (7)  5.00 (3)  4.55 (2) |
| Funding | 1  2  3 | 2.27 (2)  5.00 (3)  0.00 (0) | 9.09 (9)  20.00 (11)  13.64 (6) | 31.82 (31)  25.00 (14)  22.73 (10) | 29.55 (28)  30.00 (17)  31.82 (14) | 11.36 (11)  15.00 (9)  22.73 (10) | 13.64 (13)  5.00 (3)  9.09 (4) |
| Blueteq criteria | 1  2  3 | 15.91 (15)  10.00 (6)  18.18 (8) | 9.09 (9)  20.00 (11)  13.64 (6) | 34.09 (33)  15.00 (9)  22.73 (10) | 2.27 (2)  15.00 (9)  4.55 (2) | 9.09 (9)  5.00 (3)  9.09 (4) | 29.55 (28)  35.00 (20)  31.82 (14) |
| Commissioning issues | 1  2  3 | 9.09 (9)  5.00 (3)  9.09 (4) | 11.36 (11)  5.00 (3)  13.64 (6) | 29.55 (28)  15.00 (9)  13.64 (6) | 25.00 (24)  50.00 (29)  31.82 (14) | 6.82 (7)  10.00 (6)  13.64 (6) | 18.18 (17)  15.00 (9)  18.18 (8) |
| Unclear NICE technology appraisal wording | 1  2  3 | 4.55 (4)  15.00 (9)  13.64 (6) | 13.64 (13)  25.00 (15)  9.09 (4) | 50.00 (48)  35.00 (20)  31.82 (14) | 13.64 (13)  10.00 (6)  13.64 (6) | 2.27 (2)  5.00 (3)  4.55 (2) | 15.91 (15)  10.00 (6)  27.27 (12) |
| No barriers to use | 1  2  3 | 18.18 (17)  15.00 (9)  18.18 (8) | 15.91 (15)  15.00 (9)  22.73 (10) | 36.36 (35)  20.00 (11)  13.64 (6) | 9.09 (9)  20.00 (11)  13.64 (6) | 2.27 (2)  5.00 (3)  9.09 (4) | 11.36 (11)  15.00 (9)  18.18 (8) |

**Table 3.3.1.8:** Summarised results from Q33 of the Delphi survey, stating the top three most crucial topics for AA based on the question, “Do any of the following factors prevent you from being able to implement BAD management protocols for patients with AA, and if so, to what extent?”

| **Ref** | **Round** | **Top 3 most crucial topics for AA** |
| --- | --- | --- |
| **Q33** | 1 | 1. Available management and treatment options in your region/local area  2. Practitioner/resource capacity  3. Funding |
|  | 2 | 1. Practitioner/resource capacity  2. Available management and treatment options in your region/local area  3. Commissioning issues |
|  | 3 | 1. Funding  2. Available management and treatment options in your region/local area  3. Practitioner/resource capacity/commissioning issues |

**Table 3.3.1.9:** Results from Q36 of the Delphi survey, which asked, “Of all the topics listed in this section, which three do you feel are the most crucial to address to improve management of patients with AA?

| **Ref** | **Topic** | **Round** | **%Responses (n)** |
| --- | --- | --- | --- |
| **Q36** | Increased dermatologist awareness and training | 1  2  3 | 30.56 (29)  36.36 (21)  22.73 (10) |
|  | More available management options | 1  2  3 | 69.44 (67)  68.18 (39)  72.73 (32) |
|  | Greater prioritisation of life-limiting conditions | 1  2  3 | 25.00 (24)  18.18 (10)  31.82 (14) |
|  | Enhanced communication with patients | 1  2  3 | 50.00 (48)  40.91 (23)  45.45 (20) |
|  | Increased availability of clinical guidance | 1  2  3 | 66.67 (64)  40.91 (23)  68.18 (30) |
|  | Limitations to applying clinical guidance | 1  2  3 | 25.00 (24)  18.18 (10)  27.27 (12) |

**Table 3.3.1.10:** Summarised results from Q36 of the Delphi survey, stating the top three most crucial topics for AA based on the question, “Of all the topics listed in this section, which three do you feel are the most crucial to address to improve management of patients with AA?”

| **Ref** | **Round** | **Top 3 most crucial topics for AA** |
| --- | --- | --- |
| **Q36** | 1 | 1. More available management options 2. Increased availability of clinical guidance 3. Enhanced communication with patients |
|  | 2 | 1. More available management options 2. Increased availability of clinical guidance **and** Enhanced communication with patients |
|  | 3 | 1. More available management options 2. Increased availability of clinical guidance 3. Enhanced communication with patients |

**Table 3.3.1.11:** Results from Q48 of the Delphi survey, which asked, “In your opinion, please rate the extent to which the following factors can pose barriers when initiating new high-cost treatments (e.g., advanced systemic therapies like biologics or JAK inhibitors) for AA.”

| **Factor** | **Round** | **%Responses (n)** | | | | | |
| --- | --- | --- | --- | --- | --- | --- | --- |
|  |  | **Never** | **Rarely** | **Some-times** | **Often** | **Always** | **N/A to my practice** |
| Administrative burden (e.g., Blueteq form completion) | 1  2  3 | 0.00 (0)  0.00 (0)  0.00 (0) | 4.65 (4)  10.00 (6)  0.00 (0) | 37.21 (36)  30.00 (17)  31.82 (14) | 27.91 (27)  10.00 (6)  13.64 (4) | 13.95 (21)  20.00 (11)  13.64 (6) | 16.28 (21)  30.00 (17)  40.91 (18) |
| Medication dispensing (e.g., arranging homecare deliveries) | 1  2  3 | 0.00 (0)  10.00 (6)  0.00 (0) | 4.65 (4)  10.00 (6)  13.64 (13) | 44.19 (42)  35.00 (20)  22.73 (10) | 27.91 (27)  10.00 (6)  27.27 (12) | 6.98 (7)  20.00 (11)  4.55 (2) | 16.28 (16)  15.00 (9)  31.82 (14) |
| Local limitations (e.g., requirement to refer to a tertiary centre or block contracts) | 1  2  3 | 0.00 (0)  0.00 (0)  0.00 (0) | 4.65 (4)  20.00 (11)  4.55 (2) | 34.88 (33)  20.00 (19)  31.82 (14) | 23.26 (22)  30.00 (17)  18.18 (8) | 25.58 (25)  20.00 (11)  13.64 (6) | 11.63 (11)  10.00 (10)  31.82 (14) |
| Prescriber confidence or knowledge surrounding medication | 1  2  3 | 4.65 (4)  5.00 (3)  0.00 (0) | 6.98 (7)  15.00 (9)  18.18 (8) | 44.19 (42)  25.00 (14)  31.82 (14) | 30.23 (29)  40.00 (23)  9.09 (4) | 4.65 (4)  5.00 (3)  4.55 (2) | 6.98 (7)  10.00 (6)  36.36 (16) |
| Funding limitations | 1  2  3 | 2.33 (2)  5.00 (3)  0.00 (0) | 2.33 (2)  0.00 (0)  0.00 (0) | 37.21 (36)  35.00 (20)  31.82 (14) | 23.26 (22)  35.00 (20)  22.73 (10) | 27.91 (27)  20.00 (11)  13.64 (6) | 6.98 (7)  5.00 (3)  31.82 (14) |
| Patient reluctance/patient choice | 1  2  3 | 6.98 (7)  0.00 (0)  0.00 (0) | 23.26 (22)  25.00 (14)  13.64 (6) | 48.84 (47)  55.00 (31)  40.91 (18) | 9.30 (9)  10.00 (6)  9.09 (5) | 2.33 (2)  5.00 (3)  4.55 (2) | 6.98 (7)  5.00 (3)  31.82 (14) |
| There are no barriers | 1  2  3 | 30.23 (29)  25.00 (14)  18.18 (8) | 13.95 (13)  25.00 (14)  22.73 (10) | 18.60 (18)  10.00 (6)  13.64 (6) | 4.65 (4)  10.00 (6)  0.00 (0) | 4.65 (4)  5.00 (3)  0.00 (0) | 11.63 (11)  20.00 (11)  40.91 (18) |

**Table 3.3.1.12:** Summarised results from Q48 of the Delphi survey, stating the top three most crucial topics for AA based on the question, “In your opinion, please rate the extent to which the following factors can pose barriers when initiating new high-cost treatments (e.g., advanced systemic therapies like biologics or JAK inhibitors) for AA?”

| **Ref** | **Round** | **Top 2 main factors that cause a barrier for AA** |
| --- | --- | --- |
| **Q48** | 1 | 1. Local limitations (e.g., requirement to refer to a tertiary centre or block contracts) 2. Funding limitations |
|  | 2 | 1. Funding limitations 2. Local limitations (e.g., requirement to refer to a tertiary centre or block contracts) |
|  | 3 | 1. Funding limitations 2. Local limitations **and** Medication dispensing |

**Table 3.3.1.13:** Results from Q51 of the Delphi survey, which asked, “In the absence of 1-to-1 (including virtual) psychosocial services for patients with AA, supportive resources for patients that could be made available in the interim include [select all that apply]:”

| **Ref** | **Resource** | **Round** | **%Responses (n)** |
| --- | --- | --- | --- |
| **Q51** | Peer support groups (virtual and face-to-face) | 1  2  3 | 58.33 (56)  54.55 (31)  77.27 (34) |
|  | Topical patient association group-led webcasts/podcasts | 1  2  3 | 66.67 (64)  59.09 (34)  63.64 (28) |
|  | Patient information leaflets | 1  2  3 | 75.00 (72)  68.18 (39)  63.64 (28) |
|  | Short videos explaining treatment options | 1  2  3 | 83.33 (80)  77.27 (44)  68.18 (30) |
|  | Patient stories | 1  2  3 | 50.00 (48)  59.09 (34)  36.36 (16) |
|  | Training videos explaining risks and benefits of steroid creams | 1  2  3 | 66.67 (64)  50.00 (29)  50.00 (22) |

**Table 3.3.1.14:** Summarised results from Q51 of the Delphi survey, stating the top three most crucial topics for AA based on the question, “In the absence of 1-to-1 (including virtual) psychosocial services for patients with AA, supportive resources for patients that could be made available in the interim include [select all that apply]:”

| **Ref** | **Round** | **Most popular resources that should be made available to patients with AA in the absence of 1-to-1 psychosocial services** |
| --- | --- | --- |
| **Q51** | 1 | 1. Short videos explaining treatment options  2. Patient information leaflets  3. Topical patient association group-led webcasts/podcasts (tied)  3. Training videos explaining risks and benefits of steroid creams (tied) |
|  | 2 | 1. Short videos explaining treatment options  2. Patient information leaflets  3. Topical patient association group-led webcasts/podcasts and patient stories |
|  | 3 | 1. Peer support groups  2. Short videos  3. Topical patient association group-led webcasts/podcasts **AND** patient information leaflets |

**Table 3.3.1.15:** Results from Q55 of the Delphi survey, which asked, “Of all the topics listed in this section, which three do you feel are the most crucial to address to improve management of patients with AA?”

| **Ref** | **Topic** | **Round** | **%Responses (n)** |
| --- | --- | --- | --- |
| **Q55** | Regional variation in management/treatment pathways | 1  2  3 | 36.36 (35)  40.91 (23)  27.27 (12) |
|  | More timely availability of new treatments | 1  2  3 | 31.82 (31)  22.73 (13)  31.82 (14) |
|  | Decreased waiting times for specialist clinic appointments | 1  2  3 | 36.36 (35)  36.36 (21)  40.91 (18) |
|  | Increased funding and resource allocation | 1  2  3 | 31.82 (31)  27.27 (16)  13.64 (6) |
|  | Decreased barriers to initiating high-cost treatments | 1  2  3 | 34.09 (33)  27.27 (16)  18.18 (8) |
|  | Limitations to applying clinical guidance | 1  2  3 | 27.27 (26)  4.55 (3)  4.55 (2) |
|  | Enhanced access to psychosocial support | 1  2  3 | 22.73 (22)  18.18 (10)  18.18 (8) |
|  | Implementation of a national management pathway | 1  2  3 | 22.73 (22)  4.55 (3)  9.09 (4) |

**Table 3.3.1.16:** Summarised results from Q55 of the Delphi survey, stating the top three most crucial topics for AA based on the question, “Of all the topics listed in this section, which three do you feel are the most crucial to address to improve management of patients with AA?”

| **Ref** | **Round** | **Top 3 factors to address to improve management of patients with AA** |
| --- | --- | --- |
| **Q55** | 1 | 1. Regional variation in management/treatment pathways  2. Decreased waiting times for specialist clinic appointments  3. Decreased barriers to initiating high-cost treatments) |
|  | 2 | 1. Regional variation in management/treatment pathways  2. Decreased waiting times for specialist clinic appointments  3. Decreased barriers to initiating high-cost treatments) and Increased funding and resource |
|  | 3 | 1. Decreased waiting times for specialist clinic appointments  2. More timely availability of new treatments  3. Regional variation in management |

### SI: 3.3.2: Complete results of the Delphi survey for questions relating to AD, including responses from each survey round

**Table 3.3.2.1:** A summary of the key consensus results from the Delphi survey for AD, including all rounds of responses.

| **Ref** | **Question or statement** | **Round** | **Response % (n, round number)** | | | |
| --- | --- | --- | --- | --- | --- | --- |
|  |  |  | **Disagree** | **Neutral** | **Agree** | **N/A** |
| **Q6** | General awareness of GPs and other primary care professionals is high regarding the signs and symptoms of AD | 1 | 10.25 (10) | 12.82 (12) | 76.92 (74) | 0 (0) |
| **Q7** | The level of education and training provided to GPs and other primary healthcare professionals is sufficient for diagnosing and optimally supporting patients with AD | 1  2  3 | 30.00 (29)  27.27 (16)  27.27 (12) | 20.00 (19)  9.09 (5)  18.18 (8) | 30.00 (29)  63.64 (36)  54.55 (24) | 0 (0)  0 (0)  0 (0) |
| **Q8** | The current management in primary care is as effective as it could be for patients with AD | 1  2  3 | 33.33 (32)  40.91 (23)  31.82 (14) | 25.64 (25)  18.18 (10)  18.18 (8) | 41.03 (39)  40.91 (23)  50.00 (22) | 0 (0)  0 (0)  0 (0) |
| **Q9** | Quality disease-specific information is consistently provided by GPs to patients on possible management options and outcomes, and information on next steps for patients with AD | 1  2  3 | 35.90 (34)  31.82 (18)  36.36 (16) | 25.46 (24)  31.82 (18)  18.18 (8) | 38.46 (37)  36.36 (21)  45.45 (20) | 0 (0)  0 (0)  0 (0) |
| **Q10** | GPs appropriately refer patients to secondary care with AD | 1  2  3 | 15.38 (15)  22.73 (13)  18.18 (8) | 15.38 (15)  13.64 (8)  18.18 (8) | 69.23 (66)  63.64 (36)  63.64 (28) | 0 (0)  0 (0)  0 (0) |
| **Q11** | GP referral to secondary care is in general timely for patients with AD | 1  2  3 | 35.00 (34)  40.91 (23)  31.82 (14) | 22.50 (22)  13.64 (8)  9.09 (4) | 42.50 (41)  45.45 (26)  59.09 (26) | 0 (0)  0 (0)  0 (0) |
| **Q12** | The use of the Advice and Guidance (A&G) teledermatology portal by GPs improves the management and the process of referral to secondary care for patients with AD | 1  2  3 | 10.00 (10)  9.09 (5)  18.18 (8) | 17.50 (17)  18.18 (10)  22.73 (10) | 65.00 (62)  63.64 (36)  59.09 (26) | 7.50 (7)  9.09 (5)  0 (0) |
| **Q13** | The use of the Advice and Guidance (A&G) teledermatology portal by GPs may result in over-reliance of secondary care for conditions that could in many cases be successfully managed in primary care alone, such as AD | 1  2  3 | 38.46 (37)  22.73 (13)  45.45 (20) | 23.08 (22)  27.27 (16)  18.18 (8) | 30.77 (30)  45.45 (26)  36.36 (16) | 7.69 (7)  4.55 (3)  0 (0) |
| **Q14** | The introduction of standardised quick guides/checklists for GPs could improve primary care management of patients with AD | 1 | 2.56 (2) | 7.69 (7) | 89.74 (86) | 0 (0) |
| **Q15** | More comprehensive clinical guidelines (e.g., from the British Association of Dermatologists (BAD) and/or National Institute of Health and Care Excellence (NICE), including for specific patient populations) could improve primary care management of patients with AD | 1 | 2.56 (2) | 2.56 (2) | 94.87 (91) | 0 (0) |
| **Q17** | GPs prioritise the management of patients with AD | 1  2  3 | 25.64 (25)  31.82 (31)  13.64 (6) | 48.72 (47)  31.82 (31)  45.45 (20) | 25.64 (25)  36.36 (35)  40.91 (18) | 0 (0)  4.55 (3)  0 (0) |
| **Q19** | There is a high level of awareness and knowledge among general dermatologists (e.g., non-tertiary hair specialists) around all approved medications and their appropriate use in patients with AD | 1  2  3 | 10.53 (10)  0 (0)  9.09 (4) | 18.42 (18)  18.18 (10)  13.64 (6) | 50.00 (48)  68.18 (39)  59.09 (26) | 21.05 (20)  4.55 (3)  18.18 (8) |
| **Q20** | There is a high level of understanding of all available management strategies and their appropriate application by general dermatologists for patients with AD | 1  2 | 10.81 (10)  0 (0) | 13.51 (13)  13.64 (8) | 54.05 (52)  72.73 (41) | 21.62 (21)  4.55 (3) |
| **Q21** | There is a high level of education and training provided to general dermatologists on recognising and managing AD | 1  2  3 | 8.11 (8)  0 (0)  4.55 (2) | 10.81 (10)  31.82 (18)  18.18 (8) | 54.05 (52)  54.44 (31)  59.09 (26) | 27.03 (26)  4.55 (3)  13.64 (6) |
| **Q22** | Comprehensive and complete management is provided in secondary care for patients with AD | 1  2  3 | 8.57 (8)  9.09 (5)  18.18 (8) | 31.43 (30)  31.43 (18)  9.09 (4) | 37.41 (36)  68.18 (39)  50 (22) | 22.86 (22)  22.86 (13)  22.73 (10) |
| **Q23** | Comprehensive and complete access to care is available in secondary care for patients with | 1  2  3 | 25.71 (25)  18.18 (10)  36.36 (16) | 24.32 (23)  13.64 (8)  13.64 (6) | 31.43 (30)  54.55 (31)  31.82 (14) | 22.86 (22)  4.55 (3)  18.18 (8) |
| **Q24** | Currently available management options provided in secondary care are satisfactory for patients with AD | 1  2  3 | 21.62 (21)  13.64 (8)  13.64 (6) | 27.03 (26)  4.55 (3)  13.64 (6) | 35.14 (34)  68.18 (39)  50.00 (22) | 16.22 (16)  4.55 (3)  18.18 (8) |
| **Q25** | Although not life-threatening, clinicians prioritise management of conditions with life-limiting symptoms, such as AD | 1  2  3 | 27.03 (26)  9.09 (5)  18.18 (8) | 32.43 (31)  27.27 (16)  13.64 (6) | 27.03 (26)  50 (29)  18.18 (8) | 13.51 (13)  4.55 (3)  18.18 (8) |
| **Q26** | Quality disease-specific information is consistently provided by dermatologists to patients on possible management options and outcomes, and information on next steps for patients with AD | 1  2  3 | 10.81 (10)  0 (0)  13.64 (6) | 48.65 (47)  27.27 (16)  27.27 (12) | 18.92 (18)  59.09 (34)  40.91 (18) | 21.62 (21)  4.55 (3)  18.18 (8) |
| **Q27** | Patient-reported outcome measures are routinely used in secondary care to assess the appropriateness and success of treatments in patients with AD | 1  2  3 | 9.30 (9)  4.55 (3)  13.64 (6) | 23.26 (22)  31.82 (18)  18.18 (8) | 46.51 (45)  45.45 (26)  45.45 (20) | 20.93 (20)  9.09 (6)  22.73 (10) |
| **Q28** | Quality of life (QoL) measures/indexes are routinely used to help make treatment decisions and determine the impact on patient wellbeing for patients with AD | 1  2  3 | 11.11 (11)  13.64 (8)  27.27 (12) | 22.22 (21)  22.73 (13)  27.27 (12) | 41.67 (40)  40.91 (23)  27.27 (12) | 25.00 (25)  9.09 (5)  18.18 (8) |
| **Q30** | The guidance supplied by NICE is sufficiently comprehensive for managing patients with AD | 1  2  3 | 2.78 (3)  4.55 (3)  9.09 (4) | 41.67 (40)  27.27 (16)  18.18 (8) | 52.78 (51)  54.55 (31)  68.18 (30) | 2.78 (3)  4.55 (3)  4.55 (2) |
| **Q31** | The guidance supplied by NICE is sufficiently up to date for managing patients with AD | 1  2  3 | 11.11 (11)  9.09 (5)  18.18 (8) | 36.11 (35) 36.11 (21)  27.27 (12) | 50.00 (48)  59.09 (34)  50.00 (22) | 2.78 (3)  2.78 (2)  4.55 (2) |
| **Q35** | Additional support or guidance beyond what is currently available in the UK is not required for managing patients with AD | 1  2  3 | 43.24 (42)  45.45 (26)  63.64 (28) | 32.43 (31)  9.09 (5)  27.27 (12) | 18.92 (18)  36.36 (21)  27.27 (12) | 5.41 (5)  0 (0)  4.55 (2) |
| **Q37** | Treatment pathways are largely identical in different UK regions across the NHS for patients with AD | 1  2  3 | 22.22 (21)  9.09 (5)  18.18 (8) | 19.44 (13)  31.82 (18)  36.36 (16) | 47.22 (45)  36.36 (21)  22.73 (10) | 11.11 (11)  13.64 (8)  18.18 (8) |
| **Q38** | National guidelines (e.g., NICE guidelines or NICE technology appraisals) impact significantly on the availability and use of management options for AD | 1  2  3 | 8.33 (8)  9.09 (5)  0 (0) | 27.78 (27)  13.64 (8)  27.27 (12) | 63.89 (61)  59.09 (34)  59.09 (26) | 0 (0)  0 (0)  13.64 (6) |
| **Q39** | Following publication of NICE technology appraisals or Scottish Medicines Consortium (SMC) recommendations, there is timely implementation of new management options for patients with AD | 1  2  3 | 21.62 (21)  18.18 (10)  31.82 (14) | 40.54 (39)  18.18 (10)  31.82 (14) | 24.32 (23)  36.36 (21)  18.18 (8) | 13.51 (13)  18.18 (10)  18.18 (8) |
| **Q40** | There are no differences in access to treatment based on geographical location for patients with AD | 1  2  3 | 41.67 (40)  31.82 (18)  59.09 (26) | 36.11 (35) 18.18 (10) 22.73 (10) | 13.89 (13)  31.82 (18)  4.55 (2) | 8.33 (8)  9.09 (5)  13.64 (6) |
| **Q41** | The Quality and Outcomes Framework should set out target indicators that can be met in the management of patients with AD | 1  2  3 | 22.22 (21)  13.64 (8)  18.18 (8) | 30.56 (29)  27.27 (16)  18.18 (8) | 38.89 (37)  45.45 (26)  50.00 (22) | 8.33 (8)  4.55 (3)  13.64 (6) |
| **Q42** | The requirement to be seen in general dermatology clinics prior to a specialist dermatology clinic (if available, e.g., hair specialist clinic) can negatively impact the management of patients with AD | 1  2  3 | 22.86 (22)  40.91 (23)  27.27 (12) | 34.29 (33)  22.73 (13)  27.27 (12) | 42.86 (41)  27.27 (16)  31.82 (14) | 0 (0)  0 (0)  13.64 (6) |
| **Q43** | The waiting times from GP referral to general dermatology appointments can negatively impact access to appropriate treatment management options for patients with AD | 1 | 5.00 (5) | 2.50 (2) | 92.50 (89) | 0 (0) |
| **Q44** | The waiting times from secondary care general dermatology to tertiary care specialist appointments (e.g. tertiary hair specialists, or tertiary psychodermatology services) can negatively impact access to appropriate management options for patients with | 1 | 0 (0) | 10.81 (10) | 78.40 (75) | 10.81 (10) |
| **Q45** | Available funding and resources for dermatology services within the NHS negatively impact the management options available for patient with AD | 1 | 5.56 (5) | 8.33 (8) | 86.11 (83) | 0 (0) |
| **Q46** | Current UK public health policies and priorities limit the optimal management of AD | 1  2  3 | 8.33 (8)  13.64 (8)  9.09 (4) | 25.00 (24)  27.27 (16)  18.18 (8) | 63.89 (61)  40.91 (23)  54.55 (24) | 2.78 (3)  9.09 (5)  18.18 (8) |
| **Q47** | There is a timely and widespread access to specialist expertise in AD | 1  2  3 | 52.78 (51)  31.82 (18)  40.91 (18) | 16.67 (16)  27.27 (16)  13.64 (6) | 30.56 (29)  31.82 (18)  27.27 (12) | 0 (0)  0 (0)  13.64 (6) |
| **Q50** | Timely access to psychosocial support services is readily available for patients with AD | 1  2 | 64.86 (62)  68.18 (39) | 21.62 (21)  18.18 (8) | 8.11 (8)  9.09 (4) | 5.41 (5)  4.55 (2) |
| **Q52** | Established management pathway models, such as for psoriasis, should be adapted and implemented for an optimal patient journey for patients with AD | 1 | 0 (0) | 7.89 (8) | 89.48 (86) | 2.63 (3) |
| **Q53** | A national patient management pathway that clearly identifies all available management options and the process for selecting, initiating and monitoring is needed for patients with AD | 1 | 2.56 (2) | 12.82 (12) | 79.49 (76) | 5.13 (5) |
| **Q54** | A national patient management pathway that clearly identifies approaches and guidance for patients having an inadequate or suboptimal response to their treatment with instructions on appropriate alternative treatment options are needed for patients with AD | 1 | 4.65 (4) | 9.30 (9) | 79.10 (76) | 6.98 (7) |

**Table 3.3.2.2:** Results from Q16 of the Delphi survey, which asked whether participants were aware of incentives (e.g., financial, resource) for GPs with regards to the management of patients with AD.

| **Ref** | **Round** | **Yes** | **No** |
| --- | --- | --- | --- |
| **Q16** | 1 | 0% | 100% |

**Table 3.3.2.3:** Results from Q18 of the Delphi survey, which asked, “Of all the topics listed in this section, which three do you feel are the most crucial to address to improve management of patients with AD?”

| **Ref** | **Topic** | **Round** | **%Responses (n)** |
| --- | --- | --- | --- |
| **Q18** | Increased GP awareness and training/education | 1  2  3 | 56.41 (54)  59.09 (34)  68.18 (30) |
|  | Provision of more patient information | 1  2  3 | 51.28 (49)  40.91 (23)  40.91 (18) |
|  | Increased referral to secondary care | 1  2  3 | 7.69 (7)  13.64 (8)  18.18 (6) |
|  | Increased use of teledermatology | 1  2  3 | 30.77 (30)  27.27 (16)  36.36 (16) |
|  | Additional tools for GPs | 1  2  3 | 38.46 (37)  40.91 (23)  22.73 (10) |
|  | Improved clinical guidelines and pathways | 1  2  3 | 69.23 (66)  63.64 (36)  72.73 (32) |
|  | More incentives for GPs (e.g. financial, resource) | 1  2  3 | 15.38 (15)  18.18 (10)  22.73 (10) |

**Table 3.3.2.4:** Summarised results from Q18 of the Delphi survey, which asked, “Of all the topics listed in this section, which three do you feel are the most crucial to address to improve management of patients with AD?”

| **Ref** | **Round** | **Top 3 most crucial topics for AD** |
| --- | --- | --- |
| **Q18** | 1 | Improved clinical guidelines and pathways  Increased GP awareness and training/education  Provision of more patient information |
|  | 2 | Improved clinical guidelines and pathways  Increased GP awareness and training/education  Provision of more patient information |
|  | 3 | Improved clinical guidelines and pathways  Increased GP awareness and training/education  Provision of more patient information |

**Table 3.3.2.5:** Results from Q29 of the Delphi survey, which asked, “How familiar are you with the current NICE clinical guidelines and NICE technology appraisals for the treatment of AD.”

| **Ref** | **Round** | **%Responses (n)** | | | | | |
| --- | --- | --- | --- | --- | --- | --- | --- |
|  |  | **Not at all familiar** | **Slightly familiar** | **Somewhat familiar** | **Familiar** | **Very familiar** | **N/A to my practice** |
| **Q29** | 1  2  3 | 13.51 (13)  9.09 (5)  4.55 (2) | 2.70 (3)  9.09 (5)  18.18 (8) | 35.14 (34)  13.64 (8)  13.64 (6) | 27.03 (26)  36.36 (20)  36.36 (16) | 18.92 (18)  18.18 (10)  22.73 (22) | 2.70 (3)  4.55 (3)  4.55 (2) |

**Table 3.3.2.6:** Results from Q32 of the Delphi survey, which asked, “To what extent in your practice do you follow the British Association of Dermatologists (BAD) recommended treatment protocols and guidance for the management of patients with AD.”

| **Ref** | **Round** | **%Responses (n)** | | | | | |
| --- | --- | --- | --- | --- | --- | --- | --- |
|  |  | **Not at all** | **Rarely** | **Sometimes** | **Often** | **At all times** | **N/A to my practice** |
| **Q32** | 1  2  3 | 0.00 (0)  4.55 (3)  0.00 (0) | 8.11 (8)  9.09 (5)  0.00 (0) | 27.03 (26)  9.09 (5)  9.09 (4) | 48.65 (47)  45.45 (26)  72.73 (32) | 5.41 (5)  22.73 (13)  13.64 (6) | 10.81 (10)  0.00 (0)  4.55 (2) |

**Table 3.3.2.7:** Results from Q34 of the Delphi survey, which asked, “Do any of the following factors prevent you from being able to implement BAD management protocols for patients with AD, and if so, to what extent?”

|  | **Round** | **%Responses (n)** | | | | |
| --- | --- | --- | --- | --- | --- | --- |
|  |  | **Never** | **Rarely** | **Sometimes** | **Often** | **Always** |
| Awareness | 1  2  3 | 14.29 (14)  20.00 (11)  13.64 (6) | 28.57 (27)  40.00 (23)  40.91 (18) | 45.24 (43)  35.00 (20)  36.36 (16) | 9.52 (9)  5.00 (3)  4.55 (2) | 2.38 (2)  0.00 (0)  0.00 (0) |
| Confidence | 1  2  3 | 14.29 (14)  15.00 (9)  13.64 (8) | 33.33 (32)  40.00 (23)  40.91 (18) | 42.86 (41)  35.00 (20)  31.82 (14) | 9.52 (9)  10.00 (6)  9.09 (4) | 0.00 (0)  0.00 (0)  0.00 (0) |
| Available management and treatment options in your region/local area | 1  2  3 | 9.52 (9)  5.00 (3)  4.55 (2) | 14.29 (14)  30.00 (17)  27.27 (12) | 50.00 (48)  35.00 (20)  36.36 (16) | 23.81 (23)  25.00 (14)  22.73 (10) | 2.38 (2)  5.00 (3)  4.55 (2) |
| Practitioner/resource capacity | 1  2  3 | 7.14 (7)  0.00 (0)  9.09 (4) | 16.67 (16)  15.00 (9)  18.18 (8) | 50.00 (48)  35.00 (20)  36.36 (16) | 21.43 (21)  40.00 (23)  27.27 (12) | 4.76 (5)  10.00 (6)  4.55 (2) |
| Patient choice | 1  2  3 | 7.14 (7)  10.00 (6)  0.00 (0) | 21.43 (21)  20.00 (11)  18.18 (8) | 47.62 (46)  50.00 (29)  63.64 (61) | 19.05 (18)  15.00 (9)  13.64 (6) | 4.76 (5)  5.00 (3)  0.00 (0) |
| Implementation and adoption | 1  2  3 | 7.14 (7)  15.00 (9)  4.55 (2) | 28.57 (27)  25.00 (14)  22.73 (10) | 47.62 (46)  25.00 (14)  50.00 (22) | 14.29 (14)  25.00 (14)  18.18 (8) | 2.38 (2)  10.00 (6)  0.00 (0) |
| Ease of use | 1  2  3 | 9.52 (9)  15.00 (9)  4.55 (2) | 28.57 (27)  25.00 (14)  18.18 (8) | 47.62 (46)  40.00 (23)  68.18 (30) | 11.90 (11)  15.00 (9)  4.55 (2) | 2.38 (2)  5.00 (3)  0.00 (0) |
| Funding | 1  2  3 | 11.90 (11)  15.00 (9)  9.09 (4) | 14.29 (14)  40.00 (23)  13.64 (8) | 47.62 (46)  15.00 (9)  36.36 (16) | 26.19  25.00 (14)  27.27 (12) | 0.00 (0)  5.00 (3)  4.55 (2) |
| Blueteq criteria | 1  2  3 | 19.05 (18)  40.00 (23)  27.27 (12) | 11.90 (11)  20.00 (11)  18.18 (8) | 40.48 (39)  25.00 (14)  40.91 (18) | 7.14 (7)  10.00 (6)  9.09 (4) | 11.90 (11)  5.00 (3)  0.00 (0) |
| Commissioning issues | 1  2  3 | 9.52 (9)  25.00 (14)  9.09 (4) | 21.43 (21)  15.00 (9)  22.73 (10) | 45.24 (43)  30.00 (17)  36.36 (16) | 14.29 (14)  25.00 (14)  22.73 (10) | 7.14 (7)  5.00 (3)  4.55 (2) |
| Unclear NICE technology appraisal wording | 1  2  3 | 7.14 (7)  25.00 (14)  13.64 (6) | 26.19 (25)  25.00 (14)  31.82 (14) | 42.86 (41)  25.00 (14)  36.36 (16) | 11.90 (11)  20.00 (11)  13.64 (6 | 9.52 (9)  5.00 (3)  0.00 (0) |
| No barriers to use | 1  2  3 | 19.05 (18)  25.00 (14)  18.18 (8) | 11.90 (11)  25.00 (14)  13.64 (6) | 33.33 (32)  25.00 (14)  36.36 (16) | 14.29 (14)  15.00 (9)  18.18 (8) | 11.90 (11)  0.00 (0)  4.55 (2) |

Please note that these 4 ALL scored the exact same for often + always

**Table 3.3.2.8:** Summarised results from Q34 of the Delphi survey, which asked, “Do any of the following factors prevent you from being able to implement BAD management protocols for patients with AD, and if so, to what extent?”

| **Ref** | **Round** | **Top 3 most crucial topics for AD** |
| --- | --- | --- |
| **Q34** | 1 | 1. Available management and treatment options in your region/local area  2. Practitioner/resource capacity  3. Funding  4. No barriers to use |
|  | 2 | 1. Practitioner/resource capacity  2. Implementation and adoption  3. Available management and treatment options in your region/local area and Funding and commissioning issues |
|  | 3 | 1. Funding  2. Practitioner/resource capacity  3. Available management and treatment options in your region/local area and commissioning issues |

**Table 3.3.2.9:** Results from Q36 of the Delphi survey, which asked, “Of all the topics listed in this section, which three do you feel are the most crucial to address to improve management of patients with AD?”

| **Ref** | **Topic** | **Round** | **%Responses (n)** |
| --- | --- | --- | --- |
| **Q36** | Increased dermatologist awareness and training | 1  2  3 | 27.78 (27)  40.91 (23)  18.18 (10) |
|  | More available management options | 1  2  3 | 61.11 (59)  40.91 (23)  50.00 (29) |
|  | Greater prioritisation of life-limiting conditions | 1  2  3 | 22.22 (21)  27.27 (16)  40.91 (18) |
|  | Enhanced communication with patients | 1  2  3 | 58.33 (56)  54.55 (31)  59.09 (26) |
|  | Increased availability of clinical guidance | 1  2  3 | 55.56 (53)  31.82 (18)  50.00 (22) |
|  | Limitations to applying clinical guidance | 1  2  3 | 30.56 (29)  31.82 (18)  31.82 (14) |

**Table 3.3.2.10:** Summarised results from Q36 of the Delphi survey, stating the top three most crucial topics for AD based on the question, “Of all the topics listed in this section, which three do you feel are the most crucial to address to improve management of patients with AD?”

| **Ref** | **Round** | **Top 3 most crucial topics for AD:** |
| --- | --- | --- |
| **Q36** | 1 | 1. Available management and treatment options in your region/local area 2. Enhanced communication with patients 3. Increased availability of clinical guidance |
|  | 2 | 1. Enhanced communication with patients 2. More available management options 3. Increased availability of clinical guidance **and** Limitations to applying clinical guidance |
|  | 3 | 1. Enhanced communication with patients 2. More available management options 3. Increased availability of clinical guidance |

**Table 3.3.2.11:** Results from Q49 of the Delphi survey, which asked, “In your opinion, please rate the extent to which the following factors can pose barriers when initiating new high-cost treatments (e.g., advanced systemic therapies like biologics or JAK inhibitors) for AD.”

|  | **Round** | **%Responses (n)** | | | | | |
| --- | --- | --- | --- | --- | --- | --- | --- |
|  |  | Never | Rarely | Some-times | Often | Always | N/A to my practice |
| Administrative burden (e.g., Blueteq form completion) | 1  2  3 | 2.33 (2)  10.00 (6)  0.00 (0) | 4.65 (4)  10.00 (6)  13.64 (6) | 44.19 (42)  20.00 (11)  22.73 (10) | 18.60 (18)  20.00 (11)  13.64 (6) | 13.95 (13)  10.00 (6)  9.09 (4) | 16.28 (16)  30.00 (17)  40.91 (18) |
| Medication dispensing (e.g., arranging homecare deliveries) | 1  2  3 | 2.33 (2)  5.00 (3)  0.00 (0) | 13.95 (13)  10.00 (6)  9.09 (4) | 41.86 (40)  40.00 (23)  22.73 (10) | 16.28 (16)  20.00 (11)  27.27 (12) | 9.30 (9)  5.00 (3)  4.55 (2) | 16.28 (16)  20.00 (11)  36.36 (35) |
| Local limitations (e.g., requirement to refer to a tertiary centre or block contracts) | 1  2  3 | 2.33 (2)  5.00 (3)  0.00 (0) | 11.63 (11)  15.00 (9)  9.09 (4) | 30.23 (29)  45.00 (26)  27.27 (12) | 27.91 (27)  10.00 (6)  27.27 (12) | 16.28 (16)  10.00 (6)  4.55 (2) | 11.63 (11)  15.00 (9)  31.82 (14) |
| Prescriber confidence or knowledge surrounding medication | 1  2  3 | 4.65 (4)  15.00 (9)  4.55 (2) | 13.95 (13)  20.00 (11)  22.73 (10) | 41.86 (40)  20.00 (11)  22.73 (10) | 16.28 (16)  25.00 (14)  9.09 (4) | 11.63 (11)  5.00 (3)  4.55 (2) | 9.30 (9)  15.00 (9)  36.36 |
| Funding limitations | 1  2  3 | 0.00 (0)  10.00 (6)  4.55 (2) | 11.63 (11)  15.00 (9)  4.55 (2) | 32.56 (31)  25.00 (14)  22.73 (10) | 18.60 (18)  25.00 (14)  31.82 (14) | 25.58 (25)  10.00 (6)  4.55 (2) | 11.63 (11)  15.00 (9)  31.82 |
| Patient reluctance/ patient choice | 1  2  3 | 4.65 (4)  0.00 (0)  0.00 (0) | 13.95 (13)  25.00 (14)  22.73 (1) | 48.84 (17)  50.00 (29)  31.82 (14) | 13.95 (13)  15.00 (9)  13.64 (6) | 4.65 (4)  0.00 (0)  0.00 (0) | 11.63 (11)  10.00 (6)  31.82 (14) |
| There are no barriers | 1  2  3 | 16.28 (16)  30.00 (17)  18.18 (17) | 18.60 (18)  15.00 (9)  22.73 (1) | 20.93 (20)  15.00 (9)  9.09 (4) | 6.98 (7)  15.00 (9)  4.55 (2) | 2.33 (2)  0.00 (0)  0.00 (0) | 13.95 (13)  15.00 (9)  36.36 (16) |

**Table 3.3.2.12:** Summarised results from Q49 of the Delphi survey, stating the top three most crucial topics for AD based on the question, “In your opinion, please rate the extent to which the following factors can pose barriers when initiating new high-cost treatments (e.g., advanced systemic therapies like biologics or JAK inhibitors) for AD?”

| **Ref** | **Round** | **Top 2 main factors that cause a barrier for AD** |
| --- | --- | --- |
| **Q49** | 1 | 1. Local limitations (e.g., requirement to refer to a tertiary centre or block contracts) 2. Funding limitations |
|  | 2 | 1. Funding limitations 2. Administrative burden and Prescriber confidence or knowledge surrounding medication |
|  | 3 | 1. Funding limitations 2. Local limitations 3. Medication dispensing |

**Table 3.3.2.13:** Results from Q51 of the Delphi survey, which asked, “In the absence of 1-to-1 (including virtual) psychosocial services for patients with AD, supportive resources for patients that could be made available in the interim include [select all that apply]:”

| **Ref** | **Resources** | **Round** | **%Responses (n)** |
| --- | --- | --- | --- |
| **Q51** | Peer support groups (virtual and face-to-face) | 1  2  3 | 52.78 (51)  59.09 (34)  63.64 (28) |
|  | Topical patient association group-led webcasts/podcasts | 1  2  3 | 63.89 (61)  54.55 (31)  63.64 (28) |
|  | Patient information leaflets | 1  2  3 | 80.56 (77)  59.09 (34)  63.64 (28) |
|  | Short videos explaining treatment options | 1  2  3 | 80.56 (77)  77.27 (44)  59.09 (26) |
|  | Patient stories | 1  2  3 | 38.89 (37)  59.09 (34)  40.91 (18) |
|  | Training videos explaining risks and benefits of steroid creams | 1  2  3 | 69.44 (67)  59.09 (34)  59.09 (26) |

**Table 3.3.2.14:** Summarised results from Q51 of the Delphi survey, stating the top three most crucial topics for AD based on the question, “In the absence of 1-to-1 (including virtual) psychosocial services for patients with AD, supportive resources for patients that could be made available in the interim include [select all that apply]:”

| **Ref** | **Round** | **Most popular resources that should be made available to patients with AD in the absence of 1-to-1 psychosocial services** |
| --- | --- | --- |
| **Q51** | 1 | 1. Short videos explaining treatment options (tied)  1. Patient information leaflets (tied)  2. Training videos explaining risks and benefits of steroid creams |
|  | 2 | 1. Short videos explaining treatment options  2. Peer support groups and Patient information leaflets and Training videos and patient stories |
|  | 3 | 1. Peer support groups  2. Patient information leaflets  3. Topical podcasts |

**Table 3.3.2.15:** Results from Q55 of the Delphi survey, which asked, “Of all the topics listed in this section, which three do you feel are the most crucial to address to improve management of patients with AD?”

| **Ref** | **Factor** | **Round** | **%Responses (n)** |
| --- | --- | --- | --- |
| **Q55** | Regional variation in management/treatment pathways | 1  2  3 | 18.18 (17)  0.00 (0)  18.18 (8) |
|  | More timely availability of new treatments | 1  2  3 | 27.27 (26)  31.82 (18)  18.18 (8) |
|  | Decreased waiting times for specialist clinic appointments | 1  2  3 | 50.00 (48)  45.45 (26)  45.45 (20) |
|  | Increased funding and resource allocation | 1  2  3 | 25.00 (24)  27.27 (16)  13.64 (6) |
|  | Decreased barriers to initiating high-cost treatments | 1  2  3 | 22.73 (22)  31.82 (18)  13.64 (6) |
|  | Limitations to applying clinical guidance | 1  2  3 | 11.36 (11)  9.09 (5)  4.55 (2) |
|  | Enhanced access to psychosocial support | 1  2  3 | 25.00 (24)  45.45 (26)  31.82 (14) |
|  | Implementation of a national management pathway | 1  2  3 | 25.00 (24)  22.73 (13)  18.18 (8) |

**Table 3.3.2.16:** Summarised results from Q55 of the Delphi survey, stating the top three most crucial topics for AD based on the question, “Of all the topics listed in this section, which three do you feel are the most crucial to address to improve management of patients with AD?”

| **Ref** | **Round** | **Top 3 factors to address to improve management of patients with AD** |
| --- | --- | --- |
| **Q55** | 1 | 1. Decreased waiting times for specialist clinic appointments  2. More timely availability of new treatments  3. Increased funding and resource allocation and Enhanced access to psychosocial support and Implementation of a national management pathway (tied for 3rd place) |
|  | 2 | 1. Enhanced access to psychosocial support and Decreased waiting times for specialist clinic appointments  2. More timely availability of new treatments and Decreased barriers to initiating high-cost treatments |
|  | 3 | 1. Decreased waiting times for specialist clinic appointments  2. Enhanced access to psychosocial support |
